# Arrhythmic risk in axial spondyloarthritis: a narrative review

**DOI:** 10.1016/j.ero.2026.100219

**Published:** 2026-07-20

**Authors:** Dimitrios Bismpos, Jan Wintrich, Christian Ukena, Xenofon Baraliakos, Philipp Sebastian Lange, Philipp Sewerin

**Affiliations:** 1 Department of Cardiology/Angiology, Marien Hospital Herne, Ruhr-University Bochum, Herne, Germany; 2 Rheumazentrum Ruhrgebiet Herne, Ruhr-University Bochum, Bochum, Germany

## Abstract

Axial spondyloarthritis (axSpA) is a chronic systemic inflammatory disorder affecting the axial skeleton and potentially the peripheral joints as well as extraskeletal tissues. Chronic systemic inflammation is also known to play a major role in the pathogenesis of conduction disturbances and arrhythmias, through chronic fibrotic scarring and cytokine-mediated delay of atrioventricular (AV) conduction as well as atrial remodelling. More specifically, the prevalence of AV block appears to be high, resulting in an increased risk for pacemaker implantation. Furthermore, axSpA is associated with an increased prevalence of atrial fibrillation, even when adjusting for other cardiovascular risk predictors. As the development of arrhythmia in axSpA appears to be associated with disease activity, controlling the systemic inflammation could potentially offer a viable addition to conventional antiarrhythmic treatment. However, the possible proarrhythmic effects of conventional and newer anti-inflammatory drugs in patients with axSpA need to be further elucidated in the future.

## INTRODUCTION

Axial spondyloarthritis (axSpA), including nonradiographic axSpA and radiographic axSpA/ankylosing spondylitis (AS), is a chronic systemic inflammatory disease affecting the axial skeleton and potentially peripheral joints as well as extraskeletal tissues [1]. The extra-musculoskeletal involvement is characterised by inflammation of organs including but not limited to the uvea, gout, lungs, the nervous system, and heart [2,3]. The incidence of axSpA is reported to be 0.4 to 15 per 100,000 people per year worldwide and is described to affect men more often than women, typically at the early adolescence [4]. Although the pathogenesis of axSpA is not entirely understood, it is almost definitely immune-mediated [5], marked by the potential development of postinflammatory fibrosis [6]. The disease is genetically predisposed, as it is characterised by an association with human leukocyte antigen (HLA)-B27 [3]. The rate of positive correlation has been reported to reach up to 90% in patients with axSpA [7].

The cardiovascular manifestations of axSpA appear in 5% to 30% of patients and may include aortic regurgitation, aortitis, conduction disturbances, arrhythmias, left ventricular diastolic dysfunction, and pericarditis [1,2,[8], [9], [10], [11], [12]]. Studies have described increased rates of stroke in patients with axSpA [11,13,14], which could be partially explained by the marked prevalence of atrial fibrillation (AF) in the population [11,15]. It is important to note that cardiac involvement may be the first sign of the disease [3,9]. The pathoanatomical hallmark of the cardiovascular manifestation of the disease is fibromuscular proliferation, fibrotic scarring, and perivascular inflammatory cell infiltration in the vessels, cardiac valves, myocardium, and interventricular conduction system [12,16,17]. Of note, the presence of HLA-B27 in relation to axSpA, but even in the absence of rheumatic disease, has been linked to cardiac dysfunction [9,18].

Even though the pathogenesis of the arrhythmogenic substrate in axSpA is not entirely understood, chronic systemic inflammation is known to play a major role [19]. It is established that long-lasting inflammatory activation can lead to coronary artery disease and heart failure [20,21]. Furthermore, in the general population, inflammation has been linked with the development of arrhythmias, especially AF, through structural and electrical remodelling [22]. Of note, an association between AF and chronic inflammatory diseases, such as rheumatic disease, has already been described [23,24]. Recent studies have indicated a correlation between proinflammatory cytokines and electrophysiological changes in the myocardium, including the prolongation of cardiomyocyte action potential [25]. Indeed, inflammatory cytokines such as interleukin (IL)-1, IL-6 and tumour necrosis factor-alpha (TNF-α), leading to increased C-reactive protein, have been linked with the pathogenesis of AF [26,27]. It has been reported that the presence of those inflammatory cytokines promotes electrical remodelling of the atria through several mechanisms, such as gap junction impairment and interference in intracellular calcium-handling, causing conduction slowing and increased ectopic activity [19,28]. Moreover, proinflammatory cytokines exert electrophysiological changes in the myocardium indirectly. Considering the presence of chronic inflammation and consecutive fibrosis in axSpA, it is tempting to hypothesise that similar mechanisms could also describe the pathogenesis of arrhythmias in this population of patients. Finally, it is important to consider the proarrhythmic effects of some anti-inflammatory drugs used for the treatment of axSpA and rheumatic diseases in general.

The most consistent evidence linking axSpA to an increased risk of cardiac arrhythmias derives from studies of patients with radiographic axSpA (AS), possibly owing to the higher cumulative inflammatory burden and disease severity in this subgroup. Accordingly, studies specifically involving AS are explicitly identified throughout the manuscript when relevant. Otherwise, the term axSpA is used to denote the disease spectrum as a whole.

The aim of this review was to provide an overview of the prevalence and severity of arrhythmias and conduction disorders in patients with axSpA, as well as the arrhythmic effects of the available pharmaceutical therapy (Central Illustration).

**Central Illustration:**
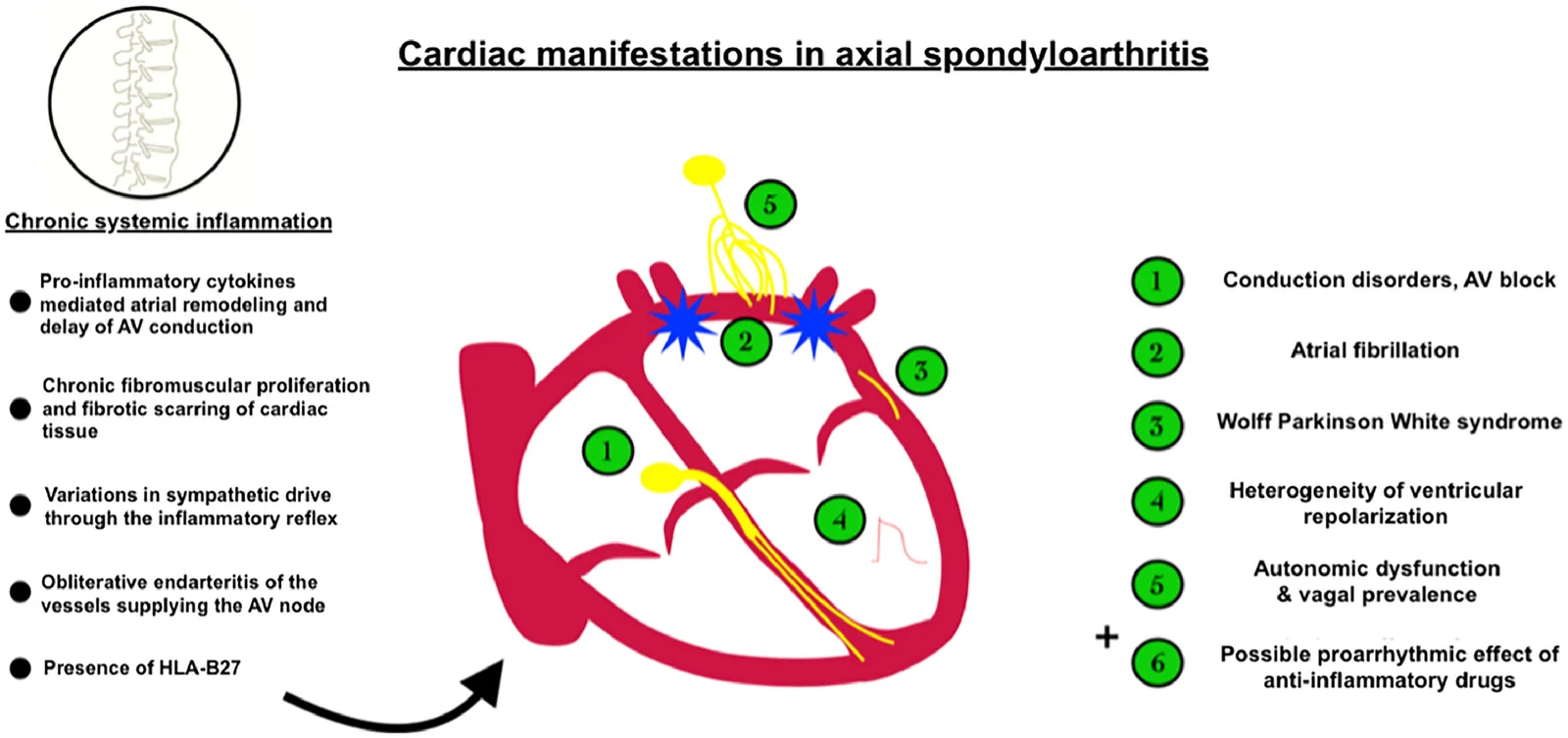
Summary of reported cardiac manifestations in axial spondyloarthritis. AV, atrioventricular; HLA, human leukocyte antigen.

## CONDUCTION SYSTEM DISORDERS

Mounting evidence suggests that systemic inflammation can delay atrioventricular (AV) conduction through cytokine-mediated effects on cardiac tissue [25,29]. In addition, inflammation could lead to destabilisation of gap junctions, as macrophages are recognised as important regulators of the AV conduction [29]. In the case of axSpA, another possible mechanism is described, as obliterative endarteritis of small vessels supplying the aortic root and AV node is a marked histological characteristic of the disease [30]. Therefore, a progression of the inflammation and fibrosis towards the AV node and bundle branches could promote conduction block [16,17]. Recent data further support this hypothesis, as a longer duration of musculoskeletal symptoms in patients with spondyloarthritis, including a substantial proportion with axSpA, was associated with an increased prevalence of both aortic root dilatation and AV block. Interestingly, the presence of enthesitis was strongly associated with the risk of AV block [31].

The prevalence of conduction system disorders varies among studies, with rates ranging from 10% to 30% having been described [18,32]. It is worthy of note that certain analyses have only reported comparable to slightly elevated rates of conduction disorders and pacemaker implantation in patients with axSpA and spondyloarthritis in general compared to the general population [[33], [34], [35]]. These findings could be explained by the partially intermittent nature of conduction disturbances in axSpA [36], as well as by limitations in the study design (eg, short follow-up period). At the same time, other investigations have reported higher rates of conduction disturbances, right bundle branch block, and AV block in axSpA [[37], [38], [39]]. Notably, Bengtsson et al [35] performed a large, nationwide cohort-based study in Sweden, examining the rates of conduction disorders among different subtypes of spondyloarthritis. They demonstrated that patients with AS (n = 6448) had a higher prevalence of AV block, resulting in a 2-fold increased risk of pacemaker implantation (Fig 1). The same study group examined patients with AS over a 5-year follow-up period, reporting an 18% rate of conduction system disorders, mainly first-grade AV block, either at baseline or at follow-up [36]. In a similar manner, a recent meta-analysis published by Morovatdar et al [40] displayed a higher risk (up to 3.5 times) for AV block occurrence in patients with AS. This observational association between AS and AV block appears to have a genetic background, as demonstrated by a mendelian randomisation analysis [41]. As mentioned above, the most consistent evidence regarding the risk for AV block is found in patients with radiographic axSpA, possibly due to the higher disease burden. Therefore, frequent monitoring for advanced conduction disturbances, even in patients with nonradiographic axSpA, appears to be sensible.

**Figure 1:**
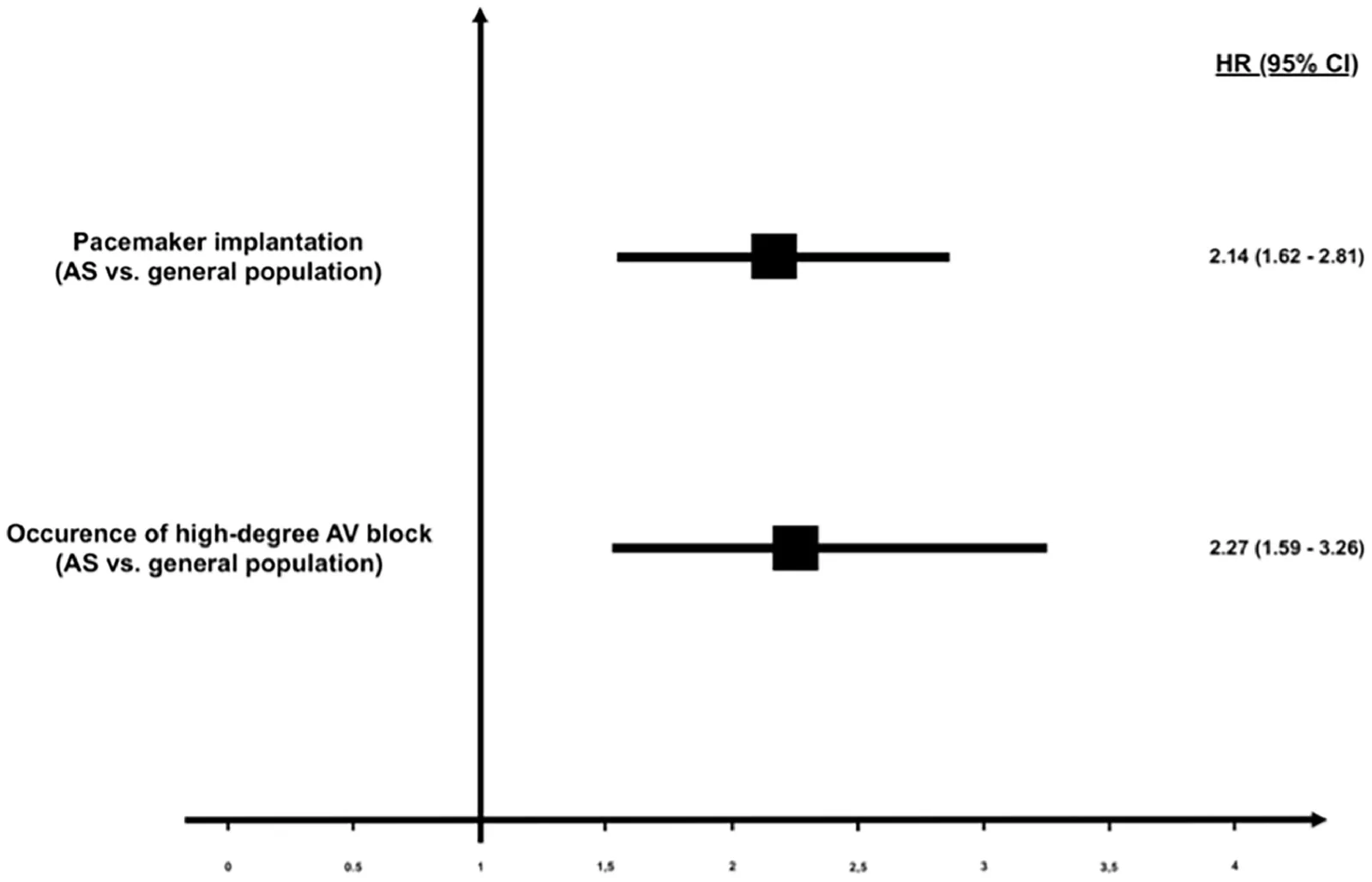
Hazard ratio for pacemaker implantation and occurrence of high-degree AV block in patients with ankylosing spondylitis compared with the general population, modified after Bengtsson et al [36]. AV, atrioventricular; AS, ankylosing spondylitis; HR, hazard ratio.

Cardiac conduction system disease in patients with axSpA correlates with age [29,36,37,42], male sex [36,37,43], and high body mass index [37]. Furthermore, patients with pre-existing lower conduction reserve appear to be more vulnerable [29]. In addition, the association between conduction disorders in axSpA and HLA-B27 has attracted interest. The presence of HLA-B27, which as previously mentioned, is linked to cardiac disease even in the absence of rheumatic disorders [18], has been associated with an increased prevalence of right bundle branch block in patients with axSpA [38]. Moreover, male patients with pacemaker-treated block have an increased frequency of HLA-B27 in rheumatic disorders [43]. In contrast with these findings, Forsblad-d’Elia et al [37] revealed no significant correlation between HLA-B27 and conduction abnormalities in patients with AS, concluding that the analysis of modern inflammatory markers may be needed to identify patients at risk. The question that therefore arises is whether disease severity begets conduction system disorders. Many studies have demonstrated a positive correlation between the occurrence of cardiac conduction abnormalities and disease duration [36,42,44] as well as the disease burden [8,36], particularly when manifested with the presence of anterior uveitis [12]. On the other hand, a correlation with peripheral arthritis does not appear to exist [36]. Of note, the analysis by Forsblad-d’Elia et al [37] failed to demonstrate an association with disease activity, possibly due to a more broadly selected cohort of patients with AS, reflective of real-world practice. In accordance with the above findings, it appears that elevated levels of C-reactive protein, erythrocyte sedimentation rate and IL-6 are associated with AV conduction abnormalities [29,44]. Apparently, systemic inflammation can worsen AV conduction not only chronically but also acutely, caused by an IL-6 mediated remodelling of the gap junctions containing connexin43 [29].

Most patients with axSpA seem to develop low-grade conduction system disorders. A prolonged PR-interval in the general population is associated with increased risk for pacemaker implantation [45], as well as AF, heart failure, and mortality [46]. Furthermore, historic data suggest that patients with axSpA and particularly AS and conduction abnormalities may have a worse prognosis [47]. Since the nature of these disturbances may be transient [36], possibly impacted by acute exacerbation of the disease [29], clinical criteria for pacemaker implantation in axSpA-related AV block remain uncertain. Therefore, in patients with active systemic inflammation and severe AV block, a targeted management of the inflammatory process, such as with anticytokine therapy, could potentially be a viable additional option [29].

## ATRIAL AND SUPRAVENTRICULAR ARRHYTHMIAS

AF is the most frequent arrhythmia in rheumatoid arthritis, commonly affecting younger, female patients [48]. Patients with rheumatoid arthritis appear to have a 30% to 40% increased risk for the development of AF compared with the general population, though a meta-analysis demonstrated a pooled risk-ratio of only 1.29 [24]. Furthermore, patients with rheumatoid arthritis have an increased risk for early AF recurrence after catheter ablation [49]. These findings are supported by studies conducted on animal models, in which atrial remodelling was associated with proinflammatory cytokines [50]. Similarly, in patients with psoriasis, investigations have found an increased risk for AF [51], caused by chronic inflammation, mediated by systemic cytokines, such as TNF-α and IL-6 [52].

Investigations regarding the prevalence, pathogenesis and severity of atrial arrhythmias in patients with axSpA are scarce in literature; however, it is intriguing to speculate that comparable mechanisms mediated by chronic, systemic inflammation could lead to the development and persistence of AF. Chronic peri- or postinflammatoric fibromuscular proliferation and fibrotic scarring may trigger increased automaticity as well as slower conduction [53], whereas the presence of HLA-B27 has been associated with the initiation of arrhythmias by increasing platelet adhesion to vessel walls [18]. Indeed, smaller studies on patients with axSpA have demonstrated delayed atriomechanical coupling intervals [54], as well as longer P-wave duration, particularly in patients with more severe disease [55].

As a result, patients with axSpA appear to have an increased risk for the development of AF, similar to rheumatoid arthritis and psoriasis, which has been demonstrated both in smaller [55] and larger patient cohorts [11,35,56]. Of note, the higher incidence of AF compared with the general population could be explained by the marked PQ-prolongation [46], which was described above. Recently, a meta-analysis conducted by Morovatdar et al [40] reported a 1.8-time higher risk for the development of AF in patients with AS. However, it has to be mentioned that these results are based on the analysis of only 3 investigations, with different diagnostic criteria for axSpA. In any case, the association between AS and AF appears to persist even after adjustment for various cardiovascular parameters [56], supporting the hypothesis that chronic inflammation begets AF (Fig 2). As mentioned above, an increased incidence of AF in patients with axSpA could certainly partly explain the higher prevalence of embolic stroke in the population [11,13,14]. As axSpA commonly develops at younger age, the risk for the development of AF in this group appears to be considerably higher, whereas the relative risk in older patients is mitigated due to coexisting cardiovascular risk factors [56]. Furthermore, a higher incidence of AF was reported in patients on TNF-inhibiting therapy, suggesting that pronounced inflammation due to higher disease activity is associated with AF [56]. These findings emphasise the need for AF screening in patients with axSpA.

**Figure 2:**
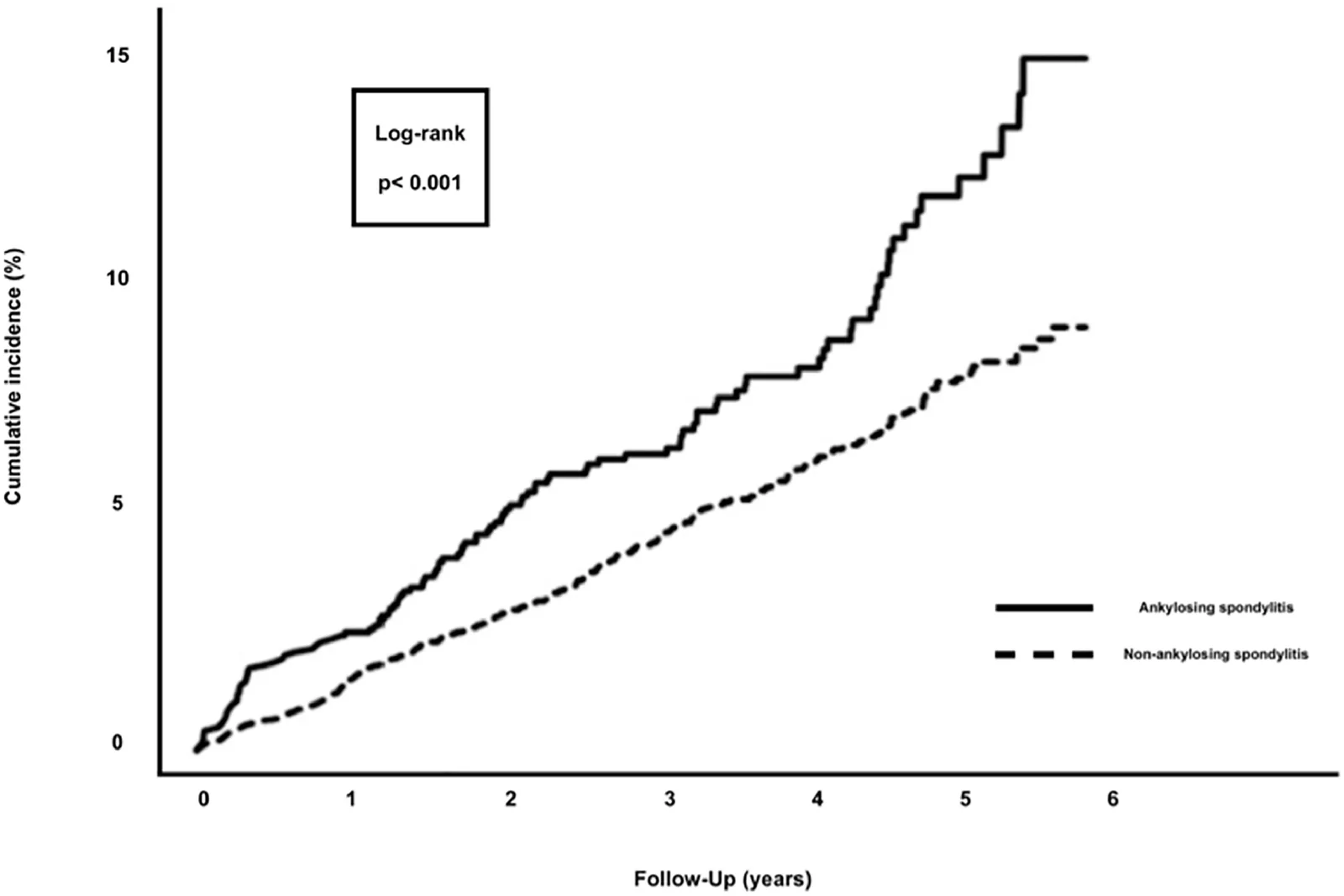
Kaplan-Meier curve for the cumulative incidence of atrial fibrillation events based on the presence of ankylosing spondylitis, modified after Moon et al [56].

It has to be noted that most of the studies mentioned above are often small scale and observational, making it difficult to distinguish causality from association. A mendelian randomisation conducted by Chen et al [57] failed to demonstrate evidence of causality between AS and AF at a genome-wide level, concluding that the observational association is most likely related to inflammation burden, medications as well as traditional risk factors. Alternative methods for elucidating the complex association between AS and AF could include local genetic correlation analysis and colocalisation analysis [57].

Moreover, the incidence of paroxysmal supraventricular tachycardias in patients with axSpA appears to be high as well. Preexcitation syndromes, namely Wolff-Parkinson-White syndrome, are reported frequently, though the pathomechanism is not yet fully understood. The prevalence of Wolff-Parkinson-White syndrome in the literature ranges from 6.24 per 1000 patients to 7.5% of patients with cardiac involvement [39,58]. As a result, patients with axSpA are more likely to develop paroxysmal supraventricular tachycardias, with a reported rate of up to 2.2% [39,57]. Of note, this subgroup of patients appears to be susceptible to the development of additional arrhythmias, including AF and atrial flutter [39]. Likewise, supraventricular paroxysmal tachycardia and Wolff-Parkinson-White syndrome appear to be frequently accompanied by signs of high disease activity, such as acute uveitis, peripheral arthritis, and bamboo spine as a sign of a long-standing and usually inadequately treated underlying disease [39].

## VENTRICULAR REPOLARISATION AND AUTONOMIC DYSFUNCTION

In the last decades, a plethora of electrocardiographic parameters have been evaluated in the general population with the aim of assessing myocardial repolarisation and the risk for ventricular arrhythmias. These include the QT interval [33], QT dispersion [59], and T peak-to-end [60]. Ventricular arrhythmias are commonly encountered in inflammatory diseases of the joints, such as rheumatoid arthritis and psoriasis [61]. Interestingly, a variety of studies have demonstrated an alteration of surrogate electrocardiographic parameters, related to chronic inflammation, which could predict the development of ventricular arrhythmias. QT dispersion was found to be prolonged in psoriasis [61], whereas similar findings have been reported in rheumatoid arthritis, especially in the presence of severe disease or elevated inflammatory parameters [19]. Therefore, it appears that chronic inflammation may have an effect on ventricular repolarisation and possibly trigger arrhythmia.

A limited number of smaller, cross-sectional studies have been conducted on patients with axSpA, reporting comparable results. Electrocardiographic parameters such as QT dispersion and T peak-to-end interval are prolonged in axSpA, the magnitude of which seems to be associated with the duration of the disease and the plasma levels of inflammatory markers, such as C-reactive protein [62]. Persistently increased systemic inflammatory activity in axSpA could lead to increased ventricular repolarisation heterogeneity, thus provoking arrhythmia, as evidenced by the increased frequency of ventricular extrasystole [62]. However, it is unclear whether these changes indicate an increased risk of ventricular arrhythmias in patients diagnosed with axSpA or simply consist of coincidental findings. The lack of larger trials on the topic, to the best of our knowledge, would probably imply the latter.

Another topic of interest is the effect of chronic inflammation in axSpA on the sympathetic tone of the heart. The impact of the autonomic nervous system on heart drive is commonly expressed by heart-rate variability [63]. A lower heart-rate variability indicates that a person is unable to adapt to microenvironmental variations [63], which has been associated with an increased risk for cardiovascular disease [64]. A number of studies have hinted at an autonomic dysfunction in patients with axSpA, indicated by lower heart-rate variability and associated with disease activity [65,66]. Recently, a meta-analysis conducted by Sharma et al [67] showed an overall decrease in heart-rate-variability parameters in patients with axSpA compared with healthy controls, which was associated with disease severity measured by the Bath Ankylosing Spondylitis Disease Activity Index (BASDAI) score. However, it has to be noted that a high heterogeneity regarding the results was observed among the included studies. These findings suggest an impairment of the cardiac autonomic nervous system in axSpA, though larger trials are needed to examine the possible clinical implications for cardiovascular outcomes.

## EFFECT OF ANTI-INFLAMMATORY DRUG THERAPY: PROARRHYTHMIC OR ANTIARRHYTHMIC?

The use of certain anti-inflammatory drugs is advised for the treatment of autoimmune and chronic inflammatory diseases, including axSpA and has changed the treatment landscape massively, resulting in significantly higher rates of remission and a remarkable improvement in the quality of life. However, their possible impact on cardiac electrophysiology is not clear and is still under investigation. It has been discussed that the therapy itself can lead through a variety of mechanisms and pathways to the development of cardiac arrhythmias, such as AF [22]. On the other hand, long-lasting disease control can reduce the inflammatory burden and therefore the arrhythmic risk [68]. To shed light on this topic, a variety of studies have been conducted on the possible arrhythmic effects of anti-inflammatory drugs on patients with chronic inflammatory arthritis. Though most studies have not focused exclusively on patients with axSpA, it has to be noted that these drugs are increasingly being used as therapeutic agents in axSpA [69].

Nonsteroidal anti-inflammatory drugs (NSAIDs) belong to the first-line therapy of axSpA [69]. Use of NSAIDs such as ibuprofen or diclofenac in commonly used doses raised the arrhythmia susceptibility in an experimental rabbit whole-heart model by shortening the effective refractory period [70]. These proarrhythmic effects could probably be mediated by inhibiting cardiac ion channels [71]. Indeed, it has been reported that the prevalence of NSAID treatment was particularly high among patients with out-of-hospital cardiac arrest [72], although exposure to conventional NSAIDs or Cyclooxygenase-2 inhibitors was not associated with an increased risk of ventricular tachycardia in a logistic regression analysis [73]. Of note, use of NSAIDs has also been linked with an increased incidence of AF [74]. This association appears to be consistent among different NSAIDs [75]. In this context, the uncertainty of potentially inadequate disease control with NSAID therapy alone must always be taken into account, which, in turn, is associated with an increased risk of arrhythmias. This is particularly evident given the historical practice of using NSAIDs for considerably extended durations, owing to the absence of viable alternatives. This has resulted in a higher disease activity being accepted over more protracted periods.

In the last decade, treatment of rheumatic diseases, such as rheumatoid arthritis and spondyloarthritis, including axSpA, has been revolutionised following the development of biologic drugs (biologic disease-modifying antirheumatic drugs [bDMARDs]), which regulate the inflammatory response by targeting specific proinflammatory cytokines and consequently the corresponding cell populations of inflammation and immunity [76]. The most common targets and accordingly the developed biologic agents include TNFa inhibitors (Infliximab, Etanercept, Adalimumab, Certolizumab, and Golimumab) and IL-17 inhibitors (Secukinumab, Ixekizumab, and Bimekizumab) [68]. Along with their beneficial impact on inflammation, a possible antiarrhythmic effect has been hypothesised. Indeed, it has been suggested that a long-lasting control of disease burden could slow the progress of a possibly coexisting structural cardiovascular disease as well as reverse the cytokine-mediated myocardial electrical instability, thus reducing the risk for arrhythmic events [68]. Furthermore, biologic agents such as TNF-α inhibitors are known to reduce cardiovascular risk by counteracting TNF-α–induced endothelial dysfunction and medial hypertrophy, processes that may also be implicated in the modulation of arrhythmogenic substrates [77]. These hypotheses are supported by a number of mostly observational studies, which have reported a rapid improvement of parameters like corrected QT interval and heart-rate variability in patients with chronic inflammatory arthritis after treatment with bDMARDs [78,79]. Of note, the use of bDMARDs, particularly IL-17 inhibitors, has been associated with a decreased risk of arrhythmia [80]. Moreover, treatment of axSpA involves today the use of targeted synthetic DMARDs, such as Tofacitinib and Upadacitinib [69]. Reassuringly, their use does not appear to be associated with increased risk for arrhythmias [81].

Simultaneously, the use of bDMARDs has also been paradoxically linked with an increased risk of cardiac arrhythmias [68]. Infliximab is a monoclonal antibody binding and neutralising TNFa, thus posing an alternative therapeutic option for patients with rheumatoid arthritis and axSpA not responding to conservative treatment [82]. Nevertheless, a plethora of case reports have suggested an increased arrhythmic risk, including bradyarrhythmias and even ventricular arrhythmias [48,63]. This was partly explained by a relative vagal prevalence in patients with chronic inflammatory arthritis receiving infliximab, as evidenced by changes in heart-rate variability [79]. In a prospective cohort of 75 patients with chronic inflammatory arthritis, Lazzerini et al [79] demonstrated that infusion with infliximab was associated with a higher incidence of ventricular arrhythmias, which tended to be more severe. Of note, these patients had prolongation of corrected QT interval and lower heart-rate variability at baseline, though these alterations were mainly seen in patients with rheumatoid arthritis, rather than spondyloarthritis. Interestingly, prolongation of the corrected QT interval, as well as the prevalence of ventricular arrhythmias, appears to subside along with the inflammatory markers as treatment continues, supporting the theory of biologic agents acutely unmasking an underlying electrophysiological instability. Finally, along with the risk for ventricular arrhythmias and bradyarrhythmias, Moon et al [56] recently reported a higher incidence of AF among patients with AS under TNFa inhibitors. However, it should be noted that infliximab’s chimeric molecular nature may be responsible for the higher reported rate of adverse events [83]. It is also noteworthy that, particularly in early descriptions, patients with a longer disease duration and, on average, permanently elevated disease activity were treated with TNF inhibitors. Consequently, the heightened risk may be partially attributed to persistent chronic inflammation and a substantially extended duration of illness, which exceeds the conventional threshold.

In conclusion, the impact of bDMARDs on patients with chronic systemic inflammatory diseases including axSpA is currently not completely clarified, as both proarrhythmic and antiarrhythmic effects have been reported, whereby the benefits of deep, sustained remission, which can be achieved much more frequently with the use of modern therapies, certainly outweigh the rather historically described potential risks. Furthermore, it has to be emphasised that although most agents are commonly used in axSpA, only a fragment of the available studies is conducted on this specific population. In addition, considering the diverse results as well as the varying methods of the conducted investigations, it could be hypothesised that the occurrence of arrhythmias is largely a sign of advanced disease, rather than a consequence of the therapy itself. Nevertheless, while a small number of mostly historical reports have suggested a possible arrhythmogenic risk of these agents, this has not been confirmed in routine clinical practice and larger prospective trials. In addition to their well-established efficacy in the management of axSpA, available data also point towards overall cardiovascular benefits, including a significant reduction in cardiovascular events [84], suggesting that the beneficial effects of these therapies may extend to arrhythmia-related outcomes.

## CONCLUSION

Through peri- and postinflammatory fibrosis- and cytokine-mediated remodelling, chronic inflammation in axSpA manifests itself in the cardiovascular system, including the pathogenesis of arrhythmias. This phenomenon is predominantly characterised by conduction system disorders as well as the induction of arrhythmias, mainly AF. Nonetheless, larger trials are needed to assess the prevalence and severity of arrhythmias in the population of axSpA as a whole, as well as in patients with elevated arrhythmic risk. As the development of arrhythmias in axSpA appears to be associated with disease severity and activity, control of the inflammatory process could potentially offer a viable alternative to conventional antiarrhythmic treatment. However, the possible proarrhythmic effects of conventional anti-inflammatory drugs in patients with axSpA need to be further elucidated in the future.

## Editor disclosure

The peer review process did not involve Editorial Board Member Xenofon Baraliakos, and the editorial decision-making was led by editors who were not involved in the creation of this manuscript.

## CRediT authorship contribution statement

**Dimitrios Bismpos:** Writing – review & editing, Writing – original draft, Supervision, Methodology, Data curation, Conceptualization. **Jan Wintrich:** Writing – original draft. **Christian Ukena:** Writing – review & editing, Supervision, Conceptualization. **Xenofon Baraliakos:** Writing – review & editing, Supervision, Conceptualization. **Philipp Sebastian Lange:** Writing – review & editing, Supervision. **Philipp Sewerin:** Writing – review & editing, Supervision, Conceptualization.

## Competing interests

The authors declare that they have no known competing financial interests or personal relationships that could have appeared to influence the work reported in this paper.
